# One hundred ways to process time, frequency, rate and scale in the central auditory system: a pattern-recognition meta-analysis

**DOI:** 10.3389/fncom.2015.00080

**Published:** 2015-07-03

**Authors:** Edgar Hemery, Jean-Julien Aucouturier

**Affiliations:** ^1^Centre de Robotique (CAOR), École Nationale Supérieure des Mines de ParisParis, France; ^2^Science et Technologie de la Musique et du Son, IRCAM/Centre National de la Recherche Scientifique UMR9912/UPMCParis, France

**Keywords:** spectro-temporal receptive fields, auditory pathway, audio pattern recognition

## Abstract

The mammalian auditory system extracts features from the acoustic environment based on the responses of spatially distributed sets of neurons in the subcortical and cortical auditory structures. The characteristic responses of these neurons (linearly approximated by their spectro-temporal receptive fields, or STRFs) suggest that auditory representations are formed, as early as in the inferior colliculi, on the basis of a time, frequency, rate (temporal modulations) and scale (spectral modulations) analysis of sound. However, how these four dimensions are integrated and processed in subsequent neural networks remains unclear. In this work, we present a new methodology to generate computational insights into the functional organization of such processes. We first propose a systematic framework to explore more than a hundred different computational strategies proposed in the literature to process the output of a generic STRF model. We then evaluate these strategies on their ability to compute perceptual distances between pairs of environmental sounds. Finally, we conduct a meta-analysis of the dataset of all these algorithms' accuracies to examine whether certain combinations of dimensions and certain ways to treat such dimensions are, on the whole, more computationally effective than others. We present an application of this methodology to a dataset of ten environmental sound categories, in which the analysis reveals that (1) models are most effective when they organize STRF data into frequency groupings—which is consistent with the known tonotopic organization of receptive fields in auditory structures -, and that (2) models that treat STRF data as time series are no more effective than models that rely only on summary statistics along time—which corroborates recent experimental evidence on texture discrimination by summary statistics.

## 1. Introduction

**Figure 1:**
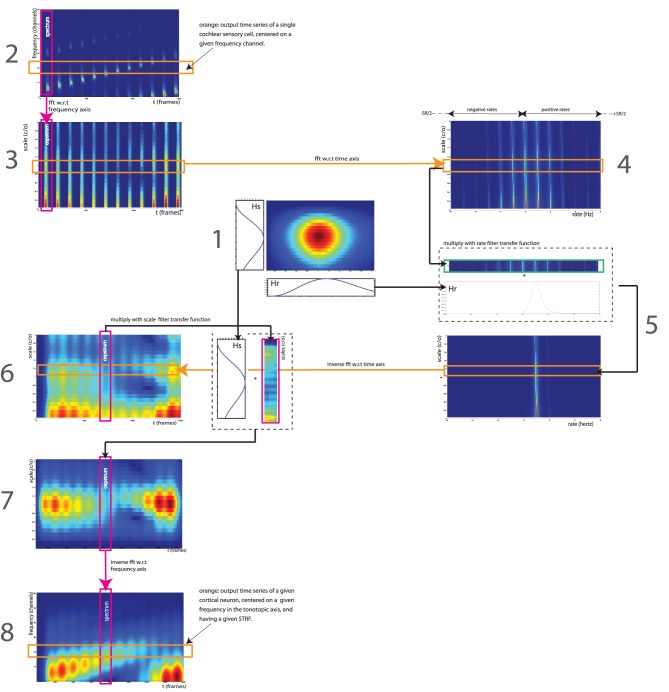
**Signal processing workflow of the STRF model, as implemented by Patil et al. (2012)**. The STRF model simulates processing occurring in the IC, auditory thalami and A1. It processes the output of the cochlea—represented here by an auditory spectrogram in log frequency (*SR* = 24 channels per octave) vs. time (*SR* = 125 Hz), using a multitude of cortical neuron each tuned on a frequency (in Hz), a modulation w.r.t time (a rate, in Hz) and w.r.t. frequency (a scale, in cycles/octave). We take here the example of a 12-s series of 12 Shepards tones, i.e., a periodicity of 1 Hz in time and 1 harmonic partial/octave in frequency, processed by a STRF centered on rate = 1 Hz and scale = 1 c/o (1). In the input representation (2), each frequency slice (orange) corresponds to the output time series of a single cochlear sensory cell, centered on a given frequency channel. In the output representation (8), each frequency slice (orange) corresponds to the output of a single auditory neuron, centered on a given frequency on the tonotopic axis, and having a given STRF. The full model (not shown here) has hundreds of STRFs (e.g., 22 rates ^*^ 11 scales = 242), thus thousands of neurons (e.g., 128 freqs ^*^ 242 STRFs = 30,976). Figure adapted from http://dx.doi.org/10.6084/m9.figshare.695010, with permission.

The mammalian auditory system extracts features from the acoustic environment based on the responses of spatially distributed sets of neurons in the inferior colliculi (IC), auditory thalami and primary auditory cortices (A1). These neurons operate on the preprocessing done by earlier subcortical nuclei such as the superior olive and cochlear nuclei, as well as the auditory periphery. The behavior of auditory neurons in IC, thalamus and, to some extent, in A1, can be modeled as a spectro-temporal filterbank, in which the transformation between the sound input and the firing-rate output of each neuron is approximated linearly by its spectro-temporal receptive field (STRF) (Chi et al., 2005). An auditory neuron's STRF can be described as a 2-dimensional filter in the space of spectro-temporal modulations, with a bandwidth in the two dimensions of rate (temporal modulation, in Hz) and scale (spectral modulation, in cycles/octave). In addition, because auditory neurons are tonotopically organized and respond to frequency-specific afferents, a given neuron's STRF only operates on a specific frequency band. The convolution between the rate-scale STRF and the time-frequency spectrogram of the sound gives an estimate of the time-varying firing rate of the neuron (Figure 1).

Although the experimental measurement of STRFs in live biological systems is plagued with methodological difficulties (Christianson et al., 2008), and their approximation of the non-linear dynamics and context-dependency of auditory (especially cortical) neurons is only partial (Gourévitch et al., 2009), computational simulations of even simple STRFs appear to provide a robust model of the representational space embodied by the central auditory system. (Patil et al., 2012) have recently demonstrated a system which uses a Gabor-filter implementation of STRFs to compute perceptual similarities between short musical tones. In their implementation, sound signals were represented as the mean output energy in time of a bank of more than 30,000 neurons, evenly spaced according to their characteristic frequencies, rates and scales. This high-dimensional representation was then reduced using principal component analysis, and used to train a gaussian-kernel distance function between pairs of sounds. The authors found that their model approximates psychoacoustical dissimilarity judgements made by humans between pairs of sounds to near-perfect accuracy, and better so than alternative models based on simpler spectrogram representation.

Such computational studies (see also Fishbach et al., 2003) provide proofs that a given combination of dimensions (e.g., frequency/rate/scale for Patil et al., 2012; frequency/rate for Fishbach et al., 2003), and a given processing applied on it, is sufficient to give good performance; they do not, however, answer the more general questions of what combination of dimensions is optimal for a task, in what order these dimensions are to be integrated, or whether certain dimensions are best summarized rather than treated as an orderly sequence. In other words, while it seems plausible that cognitive representations are formed on the basis of a time, frequency, rate and scale analysis of auditory stimuli, and while much is known about how IC, thalamus and A1 neurons encode such instantaneous sound characteristics, how these four dimensions are integrated and processed in subsequent neural networks remains unclear.

Human psychophysics and animal neurophysiology have recently cast new light on some of these subsequent processes. First, psychoacoustical studies of temporal integration have revealed that at least part of the human processing of sound textures relies only on temporal statistics, which do not retain the temporal details of the feature sequences (McDermott et al., 2013; Nelken and de Cheveigné, 2013). But the extent to which this type of timeless processing generalizes to any type of auditory stimuli remains unclear; similarly, the computational purpose of this type of representation is unresolved: does it e.g., provide a higher-level representational basis for recognition, or a more compact code for memory? Second, a number of studies have explored contextual effects on activity in auditory neurons (e.g., Ulanovsky et al., 2003, David and Shamma, 2013). These effects are evidence for how sounds are integrated over time, and constrain their neural encoding (Asari and Zador, 2009). Finally, the neurophysiology of the topological organization of auditory neuronal responses also provides indirect insights into the computational characteristics of the auditory system. For instance, it is well-established that tonotopy (the orderly mapping of characteristic frequency (CF) in space) pervades all levels of the central auditory system including subcortical nuclei such as IC (Ress and Chandrasekaran, 2013) and auditory cortex (Eggermont, 2010). This organization plausibly reflects a computational need to process several areas of the frequency axis separately, as shown e.g., with frequency-categorized responses to natural meows in cat cortices (Gehr et al., 2000). However, the topology of characteristic responses in the dimensions of rate and scale remains intriguing: while STRFs are orderly mapped in the auditory areas of the bird forebrain, with clear layer organization of rate tuning (Kim and Doupe, 2011), no systematic rate or scale gradients have been observed to date in the mammalian auditory cortex (Atencio and Schreiner, 2010, but see Baumann et al., 2011 for IC). Conversely, if, in birds, scale gradients seem to be mapped independently of tonotopy, in A1 they vary systematically within each isofrequency lamina (Schreiner et al., 2000). It is therefore plausible that the mammalian auditory system has evolved networks able to jointly process the time, frequency, rate and scale dimensions of auditory stimuli into a combined representations optimized for perceptive tasks such as recognition, categorization and similarity. But there are many ways to form such representations, and insights are lacking as to which are most effective or efficient.

This work presents a new computational approach to derive insights on what conjunct processing of the 4 dimensions of time, frequency, rate and scale makes sense in the central auditory system at the level of IC onwards. To do so, we propose a systematic pattern-recognition framework to, first, design more than a hundred different computational strategies to process the output of a generic STRF model; second, we evaluate each of these algorithms on their ability to compute acoustic dissimilarities between pairs of sounds; third, we conduct a meta-analysis of the dataset of these many algorithms' accuracies to examine whether certain combinations of dimensions and certain ways to treat such dimensions are more computationally effective than others.

## 2. Methods

### 2.1. Overview

Starting with the same STRF implementation as (Patil et al., 2012), we propose a systematic framework to design a large number of computational strategies (precisely: 108) to integrate the four dimensions of time, frequency, rate and scale in order to compute perceptual dissimilarities between pairs of audio signals.

As seen below (Section 2.2), the STRF model used in this work operates on 128 characteristic frequencies, 22 rates and 11 scales. It therefore transforms a single auditory spectrogram (dimension: 128 × time, sampled at *SR* = 125 Hz) into 22 × 11 = 242 spectrograms corresponding to each of the 242 STRFs in the model. Alternatively, its output can be considered as a series of values taken in a frequency-rate-scale space of dimension 128 × 22 × 11 = 30,976, measured at each successive time window.

The typical approach to handling such data in the field of audio pattern recognition, and in the Music Information Retrieval (MIR) community in particular (Orio, 2006), is to represent audio data as a temporal series of *features*, which are computed on successive temporal windows. Features are typically seen as points in a corresponding vector space; the series of such feature points in time represents the signal. Feature series can then be modeled and compared to one another with e.g., first-order statistical distributions (the so-called bag-of-frame approach of Aucouturier and Pachet, 2007a), dynamical models (Lagrange, 2010), Markov models (Flexer et al., 2005), or alignment distances (Aucouturier and Pachet, 2007b). Taking inspiration from this approach, we construct here twenty-six models that treat the dimension of time as a series that takes its values in various combinations of frequency, rate and scale: for instance, one can compute a single scale vector (averaged over all frequencies and rates) at each time window, then model the corresponding temporal series with a Gaussian mixture model (GMM), and compare GMMs to one another to derive a measure of distance.

However, we propose here to generalize this approach to devise models that also take series in other dimensions than time (see Sections 2.3 and 2.4). For instance, one can consider values in rate/scale space as successive steps in a frequency series (or, equivalently, successive “observations” along the frequency axis). Such series can then be processed like a traditional time series, e.g., modeled with a gaussian mixture model or compared with alignment distances. Using this logics, we can create twelve frequency-series models, twelve rate-series models and twelve scale-series models. Many of these models have never been considered before in the pattern recognition literature. Finally, we add to the list fourty four models that do not treat any particular dimension as a series, but rather apply dimension reduction (namely, PCA) on various combinations of time, frequency, rate and scale. For instance, one can average out the time dimension, apply PCA on the frequency-rate-scale space, yielding a single high-dimensional vector representation for each signal; vectors can then be compared with e.g., euclidean distance. One of these ‘vector” models happens to be the approach of (Patil et al., 2012); we compare it here with fourty-three alternative models of the same kind.

The main methodological contribution of this work does not reside in algorithmic development: while they may be applied for the first time on STRF data, none of the pattern recognition techniques used here are entirely novel. Our contribution is rather to introduce new methodology at the meta-analysis level, in particular in using inferential statistics on the performance measures of such a large set of algorithms in order to gain insights into what higher auditory stages are doing.

To do so, we propose to test each of these 108 models for its ability to match reference judgements on any given dataset of sound stimuli. For instance, given a dataset of sound files organized in categories, each of the models can be tested for its individual ability to retrieve, for any file, nearest neighbors that belong to the same category (i.e., its *precision*). The better precision is achieved by a given model, the better approximation to the actual biological processing it is taken to represent, at least for the specific dataset it is being tested on.

Finally we conduct a meta-analysis of the set of 108 precision values achieved by the models. By comparing precisions between very many models, each embedding a specific sub-representation based on the STRF space, we can generate quantitative evidence of whether certain combinations of dimensions and certain ways to treat such dimensions are, on the whole, more computationally effective than others for that dataset of sounds. For instance, among the 106 models considered here, 16 operate only on frequency, 16 on frequency and rate, and 16 on frequency and scale; if compared with inferential statistics, these 48 models provide data to examine whether there is a systematic, rather than incidental, advantage to one or the other combination.

### 2.2. STRF implementation

We use the STRF implementation of (Patil et al., 2012), with the same parameters. The STRF model simulates the neuronal processing occurring in IC, auditory thalami and, to some extent, in A1. It processes the output of the cochlea—represented by an auditory spectrogram in log frequency (*SR* = 24 channels per octave) vs. time (*SR* = 125 Hz, 8 ms time windows) using a multitude of STRFs centered on specific frequencies (128 channels, 5.3 octaves), rates (22 filters: +/−4.0, +/−5.8, +/−8.0, +/−11.3, +/−16.0, +/−22.6, +/−32.0, +/−45.3, +/−64.0, +/−90.5, +/−128.0 Hz) and scales (11 filters: 0.25, 0.35, 0.50, 0.71, 1.0, 1.41, 2.00, 2.83, 4.00, 5.66, 8.00 c/o). (Figure 1-1).

**Figure 2:**
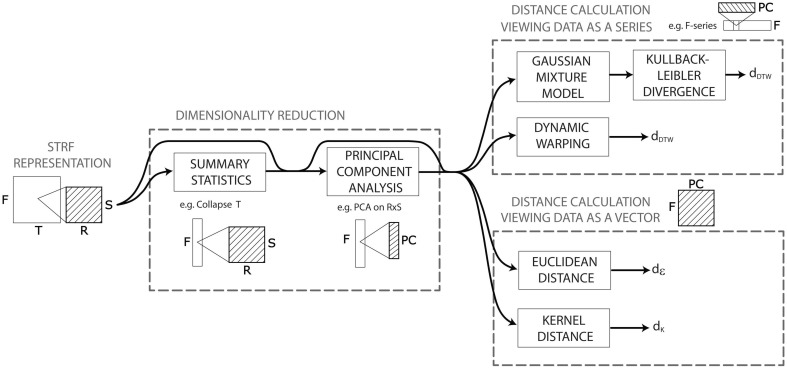
**Pattern recognition workflow of the distance calculation based on the STRF model**. The STRF model provides a high-dimensional representation upon which we construct more than a hundred algorithmic ways to compute acoustic dissimilarities between pairs of audio signals. All these algorithms obey to a general pattern recognition workflow consisting of a dimensionality reduction stage, followed by a distance calculation stage. The dimensionality reduction stage aims to reduce the dimension (*d* = 30,976 × time) of the STRF representation to make it more computationally suitable to the algorithms operating in the distance calculation stage—we use here summary statistics and/or principal component analysis (PCA). The distance computation stage differs on whether it treats a signal's STRF data as a single multidimensional point in a vector space, or as a series of points. In the former case, we use either the euclidean distance or the gaussian kernel distance. In the latter case, we use either Kullback-Leibler divergence between gaussian mixture models of the series, or dynamic programming/dynamic time warping.

**Figure 3:**
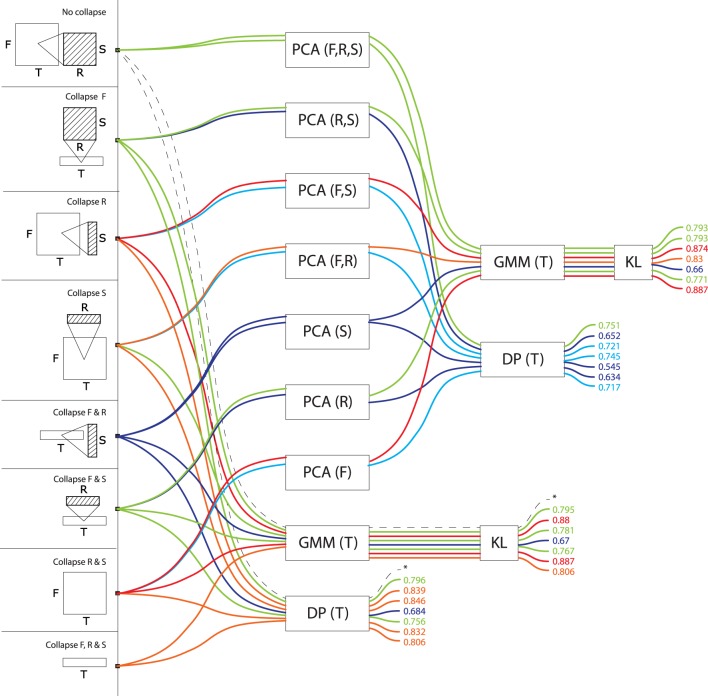
**Precision values for all computational models based on temporal series**. These models treat signals as a trajectory of features grouped by time window, taking values in a feature space consisting of frequency, rate and scale (or any subset thereof). Precisions are color-coded from blue (low, < 70%) to red (high, > 85%).

**Figure 4:**
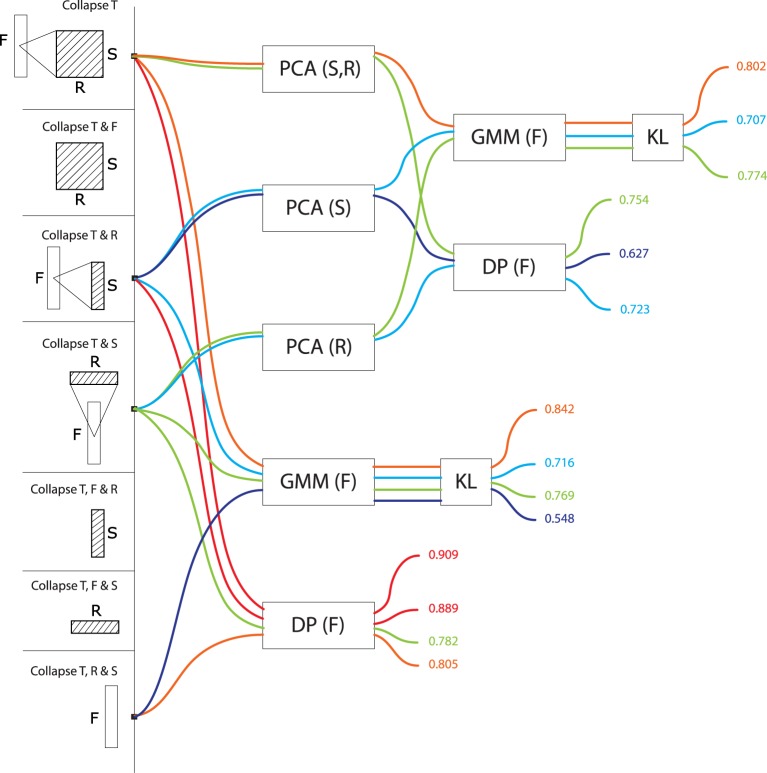
**Precision values for all computational models based on frequency series**. These models treat signals as a trajectory of values grouped by frequency, taking values in a feature space consisting of rates and scales (or any subset thereof). Precisions are color-coded from blue (low, < 70%) to red (high, > 85%).

**Figure 5:**
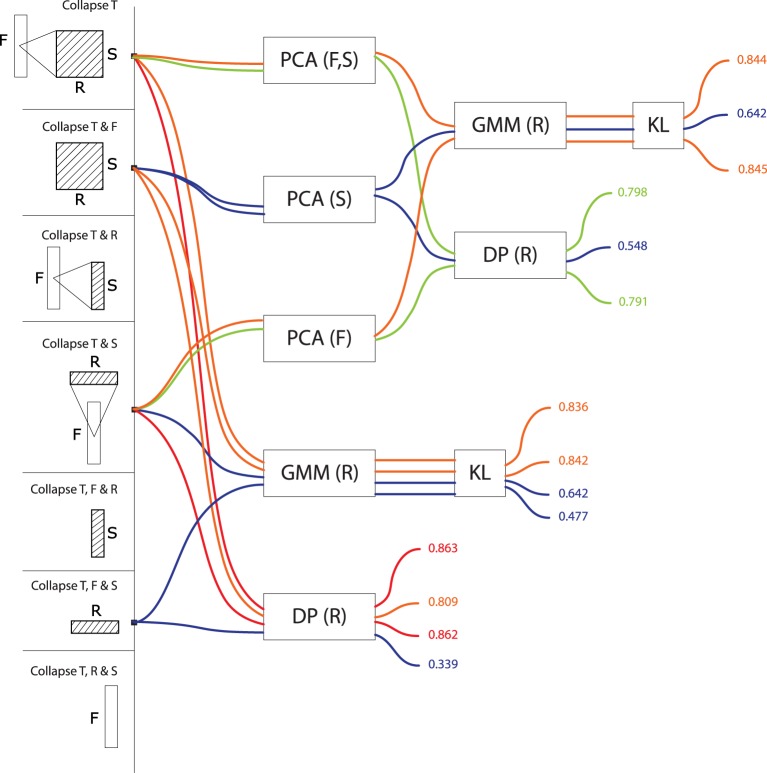
**Precision values for all computational models based on rate series**. These models treat signals as a trajectory of values grouped by rate, taking values in a feature space consisting of frequencies and scales (or any subset thereof). Precisions are color-coded from blue (low, < 70%) to red (high, > 85%).

**Figure 6:**
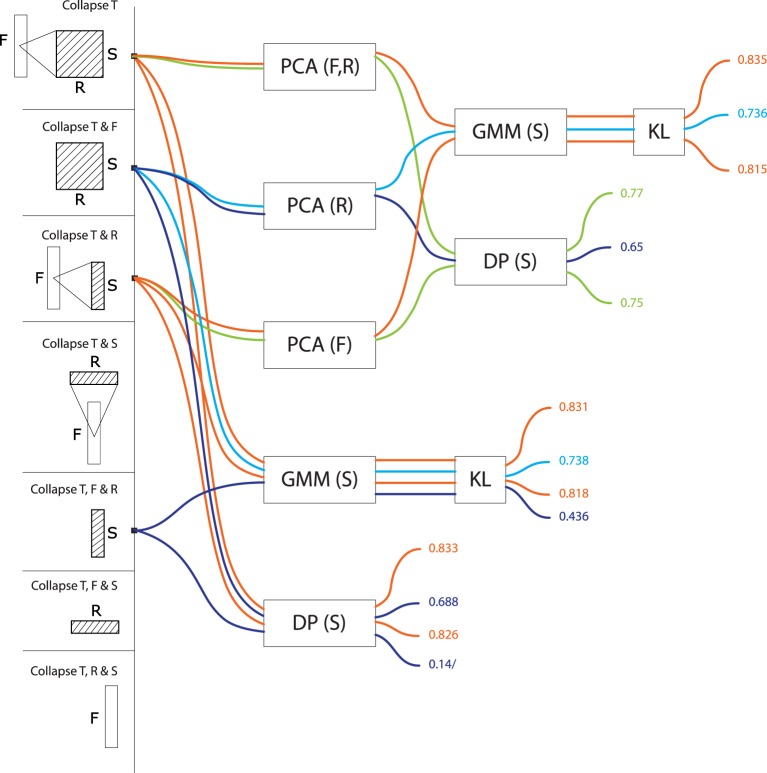
**Precision values for all computational models based on scale series**. These models treat signals as a trajectory of values grouped by scale, taking values in a feature space consisting of frequencies and rates (or any subset thereof). Precisions are color-coded from blue (low, < 70%) to red (high, > 85%).

**Figure 7:**
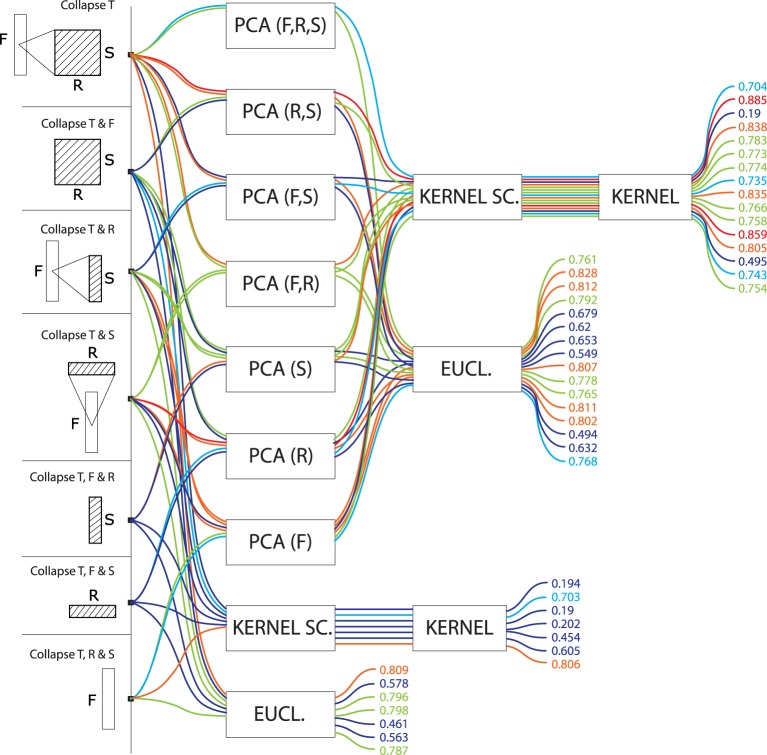
**Precision values for all computational models based on vector data**. These models do not treat any particular dimension as a series, but rather applied dimension reduction (namely, PCA) on various combinations of time, frequency, rate and scale, to yield a single high-dimensional vector representation for each signal. Vectors are compared to one another using euclidean or kernel distances. Precisions are color-coded from blue (low, < 70%) to red (high, > 85%).

**Figure 8:**
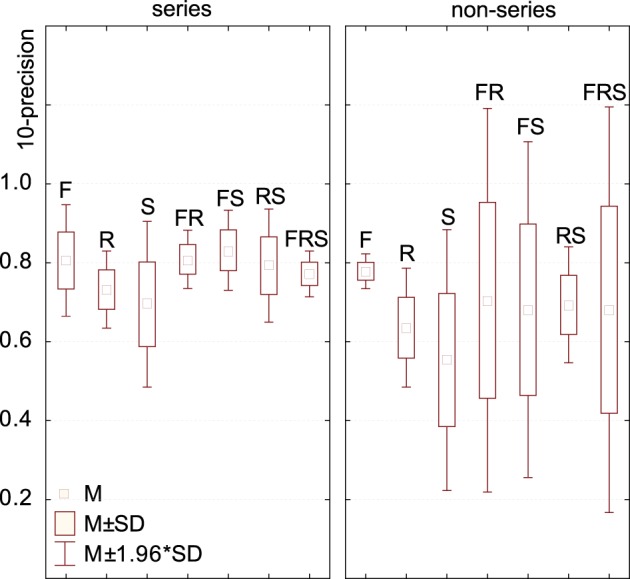
**Model performance depending on the dimensions embedded in its feature space**. For series data (regardless of the time, frequency, rate or scale basis for the series), feature spaces consisting of frequency, frequency+rate and frequency+scale were the most effective. Feature spaces consisting of only rates or scales (not in combination with frequency) were significantly less effective. For non-series data, differences were in the same trend but non-significant. Standard deviations computed over all R-precision scores achieved by all approaches using a given conjunction of dimensions as its feature space (e.g., for the series / RS group, over the precision scores of all |^*^> RS|).

Each time slice in the auditory spectrogram is Fourier-transformed with respect to the frequency axis (*SR* = 24 channels/octave), resulting in a cepstrum in scales (cycles per octave) (Figure 1-3). Each scale slice is then Fourier-transformed with respect to the time axis (*SR* = 125 Hz), to obtain a frequency spectrum in rate (Hz) (Figure 1-4). These two operations result in a spectrogram in scale (cycles/octave) vs. rate (Hz). Note that we keep all output frequencies of the second FFT, i.e., both negative rates from -SR/2 to 0 and positive rates from 0 to SR/2. Each STRF is a bandpass filter in the scale-rate space. First, we filter in rate: each scale slice is multiplied by the rate-projection of the STRF, a bandpass-filter transfer function Hr centered on a given cut-off rate (Figure 1-5). This operation is done for each STRF in the model. Each band-passed scale slice is then inverse Fourier-transformed w.r.t. rate axis, resulting in a scale (c/o) vs. time (frames) representation (Figure 1-6). We then apply the second part of the STRF by filtering in scale: each time slice is multiplied by the scale-projection of the STRF, a bandpass-filter transfer function Hs centered on a given cut-off scale (Figure 1-7). This operation is done for each STRF in the model. Each band-passed time slice is then inverse Fourier-transformed w.rt. scale axis, returning back to the original frequency (Hz) vs. time (frames) representation (Figure 1-8). In this representation, each frequency slice therefore corresponds to the output of a single cortical neuron, centered on a given frequency on the tonotopic axis, and having a given STRF. The process is repeated for each STRF in the model (22 × 11 = 242).

### 2.3. Dimensionality reduction

The STRF model provides a high-dimensional representation: (128 × 22 × 11 = 30,976) × time sampled at *SR* = 125 Hz. Upon this representation, we construct more than a hundred algorithmic ways to compute acoustic dissimilarities between pairs of audio signals. All these algorithms obey to a general pattern recognition workflow consisting of a dimensionality reduction stage, followed by a distance calculation stage (Figure 2). The dimensionality reduction stage aims to reduce the dimension (*d* = 30,976 × time) of the above STRF representation to make it more computationally suitable to the algorithms operating in the distance calculation stage and/or to discard dimensions that are not relevant to compute acoustic dissimilarities. Algorithms for dimensionality reduction can be either data-agnostic or data-driven.

1. Algorithms of the first type rely on reduction strategies that are independent of the statistical/informational properties of the specific data to which they are applied, but rather decided based on a priori, generic intuitions. As a representative example of this type of approach, we use
  - Summary statistics, in which we collapse the original STRF representation by averaging out data along one or several of its 4 physical dimensions. For instance, by averaging along time, we reduce the original time-series in a feature space of *d* = 30,976 to a single mean frame of size d:
    (1) $$STRF_{T}(f,r,s)\;=\;\frac{1}{N_{T}}\sum_{t=1}^{t=N_{T}}STRF(t,f,r,s),\forall f,r,s$$
    where *N_T_* is the number of measured time points in the original representation. By averaging along frequency, we obtain a time-series of rate-scale maps of size *d* = 22 × 11 = 242:
    (2) $$STRF_{F}(t,r,s)\;=\;\frac{1}{N_{F}}\sum_{f=1}^{t=N_{F}}STRF(t,f,r,s),\forall t,r,s$$
    where *N_F_* is the number of measured frequency points in the original representation (*N_F_* = 128).
2. Data-driven approaches to dimensionality reduction select or reorganize the dimensions of the data based on the data's specific properties, often in the aim of optimizing a criteria such as its variability or compactness. As a representative example of this approach, we use
  - Principal Component Analysis (PCA), which finds optimal linear combinations of the data's original dimensions so as to account for as much of the variability in the data as possible, while having fewer dimensions than the original. In order to compute data variability, PCA operates on the complete dataset of audio signals used for the evaluation, and then applies the optimal reduction rules on each individual signal. In this work, we implemented PCA using the fast truncated singular value decomposition (SVD) method (Halko et al., 2011), and used it to reduce the original number of dimensions to a variable number of principal components accounting for a fixed variance threshold of 99.99% of the original variance.

As illustrated in Figure 2, the two types of approaches can be applied jointly, and on any combination of dimensions. For instance, one can collapse the time dimension to create a single mean frame of size *d* = 30,976 (approach 1), then consider this collapsed data as a frequency-series (of 128 measured frequency points) taking values in the rate-scale space (*d* = 242) and apply PCA on this space to account for 99.99% of the rate-scale variance (approach 2). The result is a frequency-series (of 128 points) taking its values in a reduced feature space of dimension *d*<242.

Table 1 lists the fifteen combinations of dimensions to which the original STRF representation can be reduced. Some of these reduced representations correspond to signal representations that are well-known in the audio pattern recognition community: for instance, by averaging over frequency, rate and scale, the STRF representation is reduced to a time series of energy values, i.e., a waveform; by averaging only over rate and scale, it is reduced to a spectrogram. More sophisticated combinations are also conceptually similar to existing, if sometimes more obscure, proposals: by averaging over frequency and rate, STRF can be viewed as a time series of scale values, which is reminiscent of the Mel-frequency cepstrum coefficients that are prevalent in speech and music recognition (Logan and Salomon, 2001); time-rate representations have been previously called “modulation spectrum” (Peeters et al., 2002), and frequency-rate representations “fluctuation patterns” (Pampalk, 2006). At the other extreme, a number of reduced representations derived here from the STRF model are probably entirely original, albeit obeying to the same combinatorial framework as their better-known parents.

**Table 1:** **All possible combinations of reduced representations derived from the STRF model**.

**Dimensions**	**Summarize**	**In state-of-art as:**	**PCA possible on:**	**Processing as:**				
				**T**	**F**	**R**	**S**	**V**
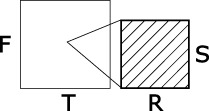	∅	STRF (Chi et al., 2005)	FRS	✓				
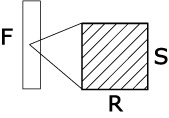	T	Average STRF maps (Patil et al., 2012)	FR, FS, FRS		✓	✓	✓	✓
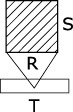	F	?	RS	✓				
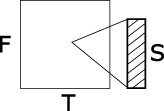	R	?	FS	✓				
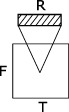	S	?	FR	✓				
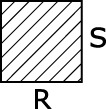	T, F	?	R, S, RS			✓	✓	✓
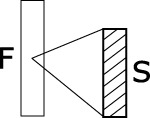	T, R	?	F, S, FS		✓		✓	✓
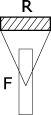	T, S	Fluctuation patterns (Pampalk, 2006)	F, R, FR		✓	✓		✓
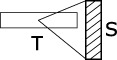	F, R	MFCCs (Logan and Salomon, 2001)	S	✓				
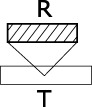	F, S	Modulation spectrum (Peeters et al., 2002)	R	✓				
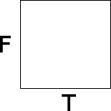	R, S	Fourier spectrogram	F	✓				
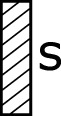	T, F, R	Average Cepstrum	S				✓	✓
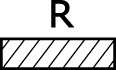	T, F, S	Periodicity transform (Sethares and Staley, 1999)	R			✓		✓
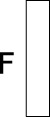	T, R, S	Fourier spectrum	F		✓			✓
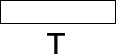	F, R, S	Waveform	∅	✓				

*Some of these reduced representations are conceptually similar to signal representations that are used in the audio pattern recognition community. We name here some which we could identify; the other unnamed constructs listed here are germane to the present study to the best of our knowledge. The choice of which distance calculation algorithm to apply on each representation depends on whether it can be as a single vector (V) or as a series in time (T), frequency (F), rate (R), or scale (S). For instance, representations in which the time dimension is preserved can only be considered as a time-series. Similarly, the combinations of dimensions that can be reduced with PCA depends on each representation. The table lists which processing is possible for each representation*.

### 2.4. Distance calculation

Following dimensionality reduction, STRF representations are compared in order to compute acoustic distances between pairs of audio signals. Distance calculation algorithms differ on whether they treat a signal's STRF data as a single multidimensional point in a vector space, or as a series of points.

1. Algorithms treating STRF data as a single multidimensional point rely on distance functions operating on the data's vector space. For the purpose of this work, we use two representative instances of such functions:
  - The simple euclidean distance, defined as
    (3) $$d_{\epsilon }(p,q)=\sqrt{\sum_{i}{\left(p_{i}-q_{i}\right)}^{2}}$$
    where *p_i_* and *q_i_* are the *i^th^* coordinate of points *p* and *q*, and
  - The gaussian kernel distance, which generalizes the approach of the euclidean distance by scaling each dimension *i* separately with a weight σ_*i*_ optimized to match the reference distance matrix we seek to obtain. It is computed as
    (4) $$d_{K}(p,\;q)=exp(-\sum_{i}\frac{{\left(p_{i}-q_{i}\right)}^{2}}{\sigma_{i}^{2}})$$
    where the σ_*i*_s are learned by gradient descent to minimize the difference between the calculated *d_K_*(*p, q*) and the true *d*(*p, q*) ∀ *p, q*, using the cost function given as:
    (5) $$J=-\frac{1}{n^{2}}\sum_{p}\sum_{q}(d(p,\;q)-\bar{d})(d_{K}(p,\;q)-{\bar{d}}_{K}$$
    where *d*(*p, q*) is the true distance between *p* and *q*, $\bar{d}$ is the mean distance over all (*p, q*) pairs, *d_K_*(*p, q*) is the kernel distance between *p* and *q* and $\bar{d}$_*K*_ is the mean kernel distance over all (*p, q*) pairs. We used the Matlab gradient descent implementation of Carl Edward Rasmussen and Olivier Chappelle (http://olivier.chapelle.cc/ams/).
2. Algorithms treating STRF data as a series of points rely on distance functions able to operate either on ordered data, or on unordered collections of points. As a representative instance of the first approach, we use:
  - The dynamic time warping (DTW) algorithm, *d_DTW_*(*p, q*), which is computed as the cost of the best alignment found between the 2 series *p* and *q*, using the individual cosine distances between all frames *p*[*n*], *n* < *length*(*p*) and *q*[*m*], *m* < *length*(*p*). Note that, if it is traditionally used with time-series, the DTW algorithm can be applied regardless of whether series *p* and *q* are ordered in time, or in any other dimension [we therefore also refer to it here by its more generic name dynamic programming (DP)]. We computed *d_DTW_* using Dan Ellis' Matlab implementation (http://www.ee.columbia.edu/~dpwe/resources/matlab/dtw/).

As a representative instance of the second approach, we use:
  - Gaussian mixture models (GMM), compared with Kullback-Leibler divergence. A GMM is a statistical model to estimate a probability distribution 𝒫(*x*) as the weighted sum of *M* gaussian distributions 𝒩_*i*_, ∀_*i*_ < *M*, each parameterized by a mean μ_*i*_ and covariance matrix Σ_*i*_,
    (6) $$𝒫(x)=\sum_{i}^{M}\pi_{i}{𝒩}_{i}(x,\mu_{i},\Sigma_{i})$$
    where π_*i*_ is the weight of gaussian distribution 𝒩_*i*_. Given a collection of points, viewed as samples from a random variable, the parameters π_*i*_,μ_*i*_,Σ_*i*_, ∀*i* < *M* of a GMM that maximizes the likelihood of the data can be estimated by the E-M algorithm (Bishop and Nasrabadi, 2006). For this work, we take *M* = 3^1^. In order to compare two series *p* and *q*, we estimate the parameters of a GMM for each of collection of points *p*[*n*] and *q*[*m*], and then compare the two GMMs 𝒫_*p*_ and 𝒫_*q*_ using the Kullback Leibler (KL) divergence:
    (7) $$d_{KL}(p,\;q)\text{ = }\int {𝒫}_{p}(x)\log\frac{{𝒫}_{q}(x)}{{𝒫}_{p}(x)}$$
    computed with the Monte-Carlo estimation method of (Aucouturier and Pachet, 2004). Note that, similarly to DTW, if GMMs, and KL divergence are traditionally used with time-series, they can be applied regardless of whether series *p* and *q* correspond to successive positions in time, or in any other dimension.
Note that, contrary to DTW, GMMs reduces a series of observations to a single random variable, i.e., discard order information: all random permutations of the series along its ordering dimension will result in the same model, while it won't with DTW distances. We still consider unordered GMMs as a “series” model, because they impose a dimension along which vectors are sampled: they model data as a collection of observations *along* time, frequency, rate or scale, and the choice of this observation dimension strongly constrains the geometry of information available to subsequent processing stages.

The choice to view data either as a single point or as a series is sometimes dictated by the physical dimensions preserved in the STRF representation after dimensionality reduction. If the time dimension is preserved, then data cannot be viewed as a single point because its dimensionality would then vary with the duration of the audio signal and we wouldn't be able to compare sounds to one another in the same feature space; it can only be processed as a time-series, taking its values in a constant-dimension feature space. For the same reason, series sampled in frequency, rate or scale cannot take their values in a feature space that incorporates time. The same constraint operates on the combination of dimensions that are submitted to PCA: PCA cannot reduce a feature space that incorporates time, because its dimensionality would not be constant. PCA can be applied, however, on the constant-dimension feature space of a time-series. Table 1 describes which modeling possibility applies to what combination of dimensions. The complete enumeration of all algorithmic possibilities yields 108 different models.

## 3. Case study: Ten categories of environmental sound textures

We present here an application of the methodology to a small dataset of environmental sounds. We compute precision values for 108 different algorithmic ways to compute acoustic dissimilarities between pairs of sounds of this dataset. We then analyse the set of precision scores of these algorithms to examine whether certain combinations of dimensions and certain ways to treat such dimensions are more computationally effective than others. We show that, even for this small dataset, this methodology is able to identify patterns that are relevant both to computational audio pattern recognition and to biological auditory systems.

### 3.1. Corpus and methods

One hundred 2-s audio files were extracted from field recordings contributions on the Freesound archive (http://freesound.org). For evaluation purpose, the dataset was organized into 10 categories of environmental sounds (*birds, bubbles, city at night, clapping door, harbor soundscape, inflight information, pebble, pouring water, waterways, waves*), with 10 sounds in each category. File formats were standardized to mono, 44.1 kHz, 16-bit, uncompressed, and RMS normalized. The dataset is available as an internet archive: https://archive.org/details/OneHundredWays.

On this dataset, we compare the performance of exactly 108 different algorithmic ways to compute acoustic dissimilarities between pairs of audio signals. All these algorithms are based on combinaisons of the four T, F, R, S dimensions of the STRF representation. To describe these combinations, we adopt the notation X>A, B… for a computational model based on a series in the dimension of X, taking its values in a feature space consisting of dimensions A, B…. For instance, a time series of frequency values is written as T>F and time series of any suitable feature space are written as T>^*^, where ^*^ is a wildcard character. In the following, PCA refers to *principal component analysis* (a data-driven dimensionality reduction method), GMM and KL to *gaussian mixture model* and *Kullback-Leibler divergence* resp. (a statistical distribution estimation method used to model series, and a distance measure used to compare models to one another), DP to *dynamic programming* (a method to compare series by computing the optimal alignment from one to the other), KERNEL SC. and KERNEL to *kernel scaling* and *kernel distance* resp. (the process of estimating optimal weights in a gaussian kernel distance with respect to a target set of dissimilarities, and the utilization of such weights to compute a distance between vectors) and EUCL to the *euclidean distance*. All these algorithms correspond to those described in Section 2.

In order to compare the performance of the algorithms, we used the same evaluation methodology as earlier work about music similarity measures (Aucouturier and Pachet, 2004): each of the models is tested for its individual ability to retrieve, for any file, nearest neighbors that belong to the same category. More precisely, for a given algorithm and a given sound query in the dataset, a result is considered relevant if the retrieved sound belongs to the same category as the query. We quantify the precision of a query using the R-precision *p_R_*, which is the precision at R-th position in the ranking of results for a query that has R relevant documents (in this case, *R* = 10):

(8) $$p_{R}=\frac{\left|\{\text{relevant documents}\}\cap \{\text{first 10 retrieved}\}\right|}{10}$$

and averaged *p_R_* over all possible queries (*n* = 100) in the test dataset to obtain a measure for each algorithm.

### 3.2. Descriptive statistics

Figures 3, 4, 5, 6, and 7 display precision scores, color-coded from blue (low, < 70%) to red (high, > 85%), for all computational models based, resp., on time-series, frequency-series, rate-series, scale-series and on the non-series, vector approach. We give here descriptive statistics in each of these five approaches. We then use inferential statistics on the complete dataset to address tranversal computational and biological questions, in the next section.

Among models that treat signals as a temporal series of features (T>^*^, Figure 3), those who incorporate frequency as one of the dimensions of the feature space tend to perform best regardless of the algorithms (DP, GMM, PCA) used to compare the series^2^. There is little advantage if any to add rates (T>F, R: precision *M* = 0.80, *SD* = 0.05, max = 0.85) or scales (T>F, S: *M* = 0.83, *SD* = 0.07, max = 0.88) to frequency only (T>F: *M* = 0.83, *SD* = 0.08, max = 0.89). Summarizing F out of the feature space is largely detrimental to precision: rates and scales alone are not effective if not linked to what frequency theyre operating on. T>R (*M* = 0.73, *SD* = 0.07, max = 0.77), T>S (*M* = 0.64, *SD* = 0.06, max = 0.68) and T>R, S (*M* = 0.76, *SD* = 0.07, max = 0.80) are all suboptimal. Among temporal series, models that compare series with GMMs (*M* = 0.80, *SD* = 0.07) tend to perform better than those who do with alignment distances (*M* = 0.74, *SD* = 0.09). Whether PCA is used or not has no effect on GMM accuracy, but it has for alignment distances: PCA: *M* = 0.67, *SD* = 0.07; no PCA: *M* = 0.79, *SD* = 0.06.

For models treating data as a frequency series (F>^*^, Figure 4), the inclusion of rates and scales in the feature vector improves precision: frequency series taking values conjunctly in rate and scale (F>S, R: *M* = 0.83, *SD* = 0.07, max = 0.91) are better than independently (F>S: *M* = 0.73, *SD* = 0.11, max = 0.89; F>R: *M* = 0.76, *SD* = 0.03, max = 0.78). Interestingly, frequency series in rate-scale space are more effective than time-series in rate-scale (T>R, S: *M* = 0.76, *SD* = 0.07, max = 0.80). There was no effect among frequency series of comparing with GMMs or alignement distance. As for temporal series, PCA had no effect on GMM algorithms, but was detrimental to alignment distances (PCA: *M* = 0.70, *SD* = 0.06; no PCA: *M* = 0.86, *SD* = 0.06).

For models treating data as a rate series (R>^*^, Figure 5) the frequency dimension is the single most effective contribution to the feature space (R>F: *M* = 0.79, *SD* = 0.10, max = 0.86; R>S: *M* = 0.71, *SD* = 0.14, max = 0.84). The conjunct use of F and S improves performance even further: R>F, S: *M* = 0.84, *SD* = 0.03, max = 0.86. The performance of R>F, S is in same range as T>F, S (*M* = 0.83, *SD* = 0.07, max = 0.88), and T>F (*M* = 0.83, *SD* = 0.08, max = 0.89). There was no effect among rate series of using either GMMs or alignment distances (GMM: *M* = 0.77, *SD* = 0.10 vs. DP: *M* = 0.77, *SD* = 0.11). As above, there was no effect of PCA on GMM performance (PCA: *M* = 0.77, *SD* = 0.11; no PCA: *M* = 0.77, *SD* = 0.11), but it was detrimental to alignment distances: PCA: *M* = 0.71, *SD* = 0.14; no PCA: *M* = 0.84, *SD* = 0.03.

Scale-series (S>^*^, Figure 6) in frequency space (S>F: *M* = 0.80, *SD* = 0.04, max = 0.83) are better than in rate space (S>R, *M* = 0.70, *SD* = 0.04, max = 0.74), and only marginally improved by combining rate and frequency (S>FR, *M* = 0.82, *SD* = 0.03, max = 0.83). For rate series, GMMs tend to be more effective than alignment distances (GMM: *M* = 0.80, *SD* = 0.05; DP: *M* = 0.75, *SD* = 0.07). As above, there was no effect of PCA on GMM accuracy, and a detrimental effect of PCA on alignment distances (PCA: *M* = 0.72, *SD* = 0.06; no PCA: *M* = 0.78, *SD* = 0.08).

Finally, models which did not treat data as a series, but rather as a vector data (Figure 7) performed generally worse (*M* = 0.68, *SD* = 0.18) than models treating data as series (*M* = 0.77, *SD* = 0.08). There was no clear advantage to any conjunction of dimensions for these models. Euclidean distances were more effective (*M* = 0.71, *SD* = 0.11) than kernel distances (*M* = 0.65, *SD* = 0.23). PCA had no strong effect on the former (PCA: *M* = 0.72, *SD* = 0.10; no PCA: *M* = 0.68, *SD* = 0.14) but was crucial to the latter (PCA: *M* = 0.73, *SD* = 0.16; no PCA: *M* = 0.45, *SD* = 0.26).

### 3.3. Computational and biological inferences from data

We use here inferential statistics to show how this set of precision scores can be used to give insights into questions related to computational and biological audio systems. In all the following, performance differences between sets of algorithms were tested with one-factor ANOVAs on the R-precision values, using various algorithmic properties as a between-subject factor.

1. **Are STRF representations more effective than spectrograms?**
  The results of (Patil et al., 2012) were taken to indicate that the modulation features (rates and scales) extracted by STRFs are crucial to the representation of sound textures, and that the simpler, and more traditionally used, time-frequency representations are insufficient both from a computational and biological point of view. Data from the above case-study, based on more than a hundred alternative algorithms, provides more contrasted evidence.
  In order to link performance to the conjunction of dimensions used in the models' feature space, we performed a one-factor ANOVA using a 6-level dimension factor: R, S, R, F-S, F-R, and F-S-R. For series data (regardless of the time, frequency, rate or scale basis for the series), there was a main effect of dimension: *F*_(6, 55)_ = 4.85, *p* = 0.0005. *Post-hoc* difference (Fisher LSD) revealed that both ^*^>R and ^*^>S feature spaces are significantly less effective than ^*^>F, ^*^>RS and any combination of F with S, R. (Figure 8). For vector data, there was no main effect of dimension: *F*_(6, 37)_ = 0.51, *p* = 0.79.
  In other words, processing the rate and scale dimensions only benefits algorithms which also process frequency, and is detrimental otherwise. Moreover, algorithms which only process frequency are no less effective, for the task and corpus of the present case-study, than algorithms which also process rate and scale.
  It is still possible that, because of their sparser nature, scale and rate representations allow faster, rather than more effective, responses that the more redundant time-frequency representations, as do efficient coding strategies in the visual pathway (Serre et al., 2007). Second, such representations may also be more learnable, e.g., requiring fewer training instances to build generalizable sensory representations.
2. **Is any model introduced here better than STRFs or spectrograms?**
  In our framework, the STRF approach implemented by (Patil et al., 2012) can be described as non-series (“summarize T”), with PCA on the 30,976-dimension F-R-S space, then a kernel distance (the top-most path in Figure 7). On our dataset, this approach lead to a R-precision of 70%.
  Among the 105 other models tested in the present study, some were found more effective for our specific task: if keeping with non-series models, a simple improvement is to apply PCA only on the 22-dimension R-S space while preserving the 128 dimensions of the frequency axis (88% R-precision). More systematically, better results were achieved when considering data as a series rather than a vector. For instance, modeling the time dimension as a GMM rather than a one-point average, otherwise keeping the same feature space and PCA strategy yields an improvement of 10% (79.3%, top-most path in Figure 3).
  Incidentally, the best results obtained on our dataset were with a rather uncommon frequency-series approach, modeling frequency-aligned observations in rate-scale space (F>R, S) with DTW (i.e., *modulation-spectrum dynamic frequency warping*). The approach lead to a R-precision of 91%.
3. **Is PCA-based dimensionality reduction a good idea with STRFs?**
  PCA dimensionality reduction was tested both for series (with GMM and alignment distances) and for non-series models (with euclidean and kernel distances). Its effect on precision was surprisingly algorithm-dependent. For series models based on GMM modeling, PCA had no statistical effect on performance as tested by ANOVA: *F*_(1, 14)_ = 0.00001, *p* = 0.99. However, using PCA was significantly detrimental when series were compared with alignment distances: *F*_(1, 14)_ = 46.932, *p* = 0.00001, with a 11% drop of R-precision (PCA: *M* = 0.70, *SD* = 0.08; no PCA: *M* = 0.81, *SD* = 0.06). Similarly, for non-series models, PCA had no effect on euclidean distance: *F*_(1, 21)_ = 0.49, *p* = 0.48 (PCA: *M* = 0.72, *SD* = 0.10; no PCA: *M* = 0.68, *SD* = 0.14), but it was crucial to the good performance of kernel distances: *F*_(1, 21)_ = 9.63, *p* = 0.005, with a 28% increase of R-precision (PCA: *M* = 0.73, *SD* = 0.16; no PCA: *M* = 0.45, *SD* = 0.26).
  From a computational point of view, such mixed evidence does not conform to pattern-recognition intuition: data-driven dimensionality reduction is a standard processing stage after feature extraction (Müller et al., 2011) and efficient coding strategies are often directly incorporated in features themselves (e.g., discrete cosine transform in the MFCC algorithm—Logan and Salomon, 2001). The detrimental impact of PCA on alignement distances may be a consequence of the whitening part of the algorithm, which balances variance in all dimensions and does not not preserve the angles/cosine distances between frame vectors; whitening has no predicted consequence on GMMs, the covariance matrices of which can scale to compensate.
  From a biological point of view, that PCA-like processing should be of little effect if applied to STRF suggests, first, that the STRF representation extracted by IC neurons onwards is already the result of efficient coding. This confirms previous findings that codewords learned with sparse coding strategies over speech and musical signals loosely correspond to the STRFs elicited with laboratory stimuli (Klein et al., 2003). Second, this suggests that subsequent processing that operates on the STRF layers in IC, thamali and A1 does not so much generate generic and efficient representations based on STRF, but perhaps rather act as an associative level that groups distributed STRF activations into intermediate and increasingly specific representations - eventually resulting in cortical specializations such as the lateral distinctions between fast and slow features of speech prosody in the superior temporal gyri (Schirmer and Kotz, 2006).
4. **Are we right to think in time (-series)?**
  All algorithms considered, models than treat signals as a series of either T, F, R, or S tend to perform better (*M* = 0.77, *SD* = 0.08) than models that are solely based on summary statistics (*M* = 0.68, *SD* = 0.18), *F*_(1, 108)_ = 13.04, *p* = 0.00046. However, among series, there was strikingly no performance advantage to any type of series: *F*_(3, 60)_ = 0.02, *p* = 0.99 (T-series: *M* = 0.77, *SD* = 0.08; F-series: *M* = 0.77, *SD* = 0.08; R-series: *M* = 0.78, *SD* = 0.10; S-series: *M* = 0.77, *SD* = 0.06). In particular, there was no intrinsic advantage to the traditional approach of grouping features by temporal windows. Further, the best results obtained in this study were with a frequency series (F>R, S with DTW).
  From a computational point of view, this pattern is in stark contrast with the vast majority of audio pattern recognition algorithms that model signals as temporal series. A wealth of recent research focuses on what model best accounts for the temporal dynamics of such data, comparing statistical mixtures over time (Aucouturier and Pachet, 2007a) with e.g., Markov models (Flexer et al., 2005), explicit dynamical models (Lagrange, 2010) or multi-scale pooling (Hamel et al., 2011). Our results suggest that collapsing the temporal dimension does not necessarily lead to reduced performance; what seems to matter rather is to group feature observations according to *any* physical dimensions of the signal, e.g., frequency. Such alternative, non-temporal paradigms remain mostly unexplored in the audio pattern recognition community.
  From a biological point of view, this pattern suggests that, for the task studied here, structured temporal representations are not a computational requirement. This is compatible with recent experimental evidence showing that at least part of the human processing of sound textures relies only on summary statistics (McDermott et al., 2013; Nelken and de Cheveigné, 2013).
5. **Does the topology of neuronal responses determine cortical algorithms?**
  The orderly mapping in cortical space of characteristic neuronal responses, such as the tonotopical map of characteristic frequencies, plausibly reflects a computational need to process several areas of the corresponding dimensions conjunctly (Eggermont, 2010). Performance data for the group of algorithms investigated in this study seems to corroborate this intuition. First, the most efficient models for our task tend to operate primarily on frequency: rate and scale data is only effective if treated conjunctly with frequency, and it can be summarized out to little cost as long as the frequency axis is maintained (Figure 8). Second, in F-R-S models, it was found more effective to reduce the dimensionality of the R-S space while preserving the F axis, rather than reducing the dimension of the conjunct F-R-S space (Figure 7). Third, the best performing algorithm found here treats data as a frequency series, i.e., a series of successive R-S maps measured along the tonotopical axis (F>RS). Finally, models that put similar emphasis on R and S rather than F are typically low performers, and processing either R and S appears to be relatively inter-changeable. This computational behavior therefore fully supports a structurative role of the frequency dimension in brain representations of sound, and is in accordance with the fact that no rate and scale gradients have been observed to date in the mammalian auditory cortex, even within each isofrequency lamina (Atencio and Schreiner, 2010).
6. **What are the neuronal equivalents of the series and vector approaches, and why is the former more effective?**
  Contrary to the vector approach, series models proceed by grouping feature observations in successive (if time-based) or simultaneous (if frequency-, rate- or scale-based) categories, providing a two-layer representation of the data. All algorithms considered, such representations (^*^>^*^) appear more effective (*M* = 0.77, *SD* = 0.08) than those which treat STRF data as a single unstructured ensemble (*M* = 0.68, *SD* = 0.18), *F*_(1, 108)_ = 13.0, *p* = 0.0004. While this computational observation is in some accordance with the tonotopic organization of auditory structures, it is unclear why it should be more effective. First, grouping STRF activation data into several categories that can be considered simultaneously may be a simple and agnostic way to represent heterogeneous stimuli, e.g., stimuli that are slowly-changing in the low-frequency band while rapidly-changing in the high-frequency band (Lu et al., 2001). Second, such structured representations may provide a more compact code for storing exemplars in memory (McDermott et al., 2013). This may further indicate that the memory structures that store sensory traces for e.g., exemplar comparison, are organized in the same structured laminae as the sensory structures—see also (Weinberger, 2004).
  Additionally, to process such series data, there was no strong difference between the GMM and DP approaches: GMMs yielded marginally superior performance for time- and scale-series and were equivalent to DP for frequency- and rate-series. This computational observation suggests that, while it is important to group data into categories, there is no strong requirement to process the differences/transitions from one category to the next (as done by DP); rather, it is the variability among categories (as modeled by GMMs) that seems most important to account for.

## 4. Discussion and generalizability

Meta-analysis of the precision values in the above case-study revealed that the most effective representations to retrieve the categorical structure of the corpus should (1) preserve information about center frequency rather than averaging over this dimension, and (2) process the output as a series, e.g., with respect to this center-frequency dimension and not necessarily to time. These two computational trends are in interesting accordance with the tonotopical organization of STRFs in central auditory structures (Eggermont, 2010; Ress and Chandrasekaran, 2013) as well as recent findings on texture discrimination by summary statistics (McDermott et al., 2013; Nelken and de Cheveigné, 2013). More generally, this suggests that meta-analysis over a space of computational models (possibly explored exhaustively) can generate insights that would otherwise be overlooked in a field where current results are scattered, having been developed with different analytical models, fitting methods and datasets.

We designed the space of computational models analyzed in the present case-study to explore the specific issue of dimension integration and reduction, in an attempt to generalize claims that, e.g., FRS representations were always better than F. As such, our analysis leaves out a number of other computational factors that may both have an impact on model performance and be generative of biological insights into what real auditory systems are doing. One of these factors is the summarization strategy used to integrate dimensions which, in this work, is fixed to the MEAN operator. We based our choice of MEAN on pilot data (all possible collapses of FRS, compared with Euclidean Distance, i.e., bottom-most stream of paths in Figure 7), for which it was systematically better than max, min and median. However, we cannot exclude that other operators than mean, incl. max, min, median, and more generally all statistical moments, would achieve better performance in the other algorithmic paths tested in the paper, or in other tasks and types of stimuli. In particular, there has been some recent convergence in the convolutional vision model literature over max-pooling, which seems to out-perform average-pooling when data sparsity is high (Boureau et al., 2010), and appears closer to physiology in the cat primary visual cortex (Lampl et al., 2004).

Similarly, our methodology may be generalized to consider other aspects of algorithmic behavior than categorization accuracy (as calculated here with R-precision), e.g., information loss, processing speed or representation compactness. While we did not find here a systematic effect of rate and scales on precision, it is possible that these physical dimensions have a beneficial impact on these other performance metrics, making them valuable features of the biological systems, e.g., helping reducing memory or attention load, and processing speed (see a similar discussion in McDermott et al., 2013).

More critically, the generalizability of results from the present case-study depends critically on both the representativity of the corpus (here, a relatively small subset of environmental sounds) and the relevance of the task (sound source categorization). It is well-known that pattern recognition methods (both in terms of feature representation, classifiers or distance metrics) depend critically on the structure of the data itself, e.g., how many exemplars and how much variance in each category, as well as how much overlap between categories (see e.g., Lagrange et al., 2015 for a recent case of this going wrong). The corpus used here results of a compromise between the need to reflect the full range of natural sounds (e.g., bird songs and water textures) and the need to include overlapping categories (e.g., pouring water and waterways). However, it remains difficult to assess the extent conclusions from the present case-study may simply reflect the specific structure of the sounds and task used in the analysis. For instance, the importance of preserving center frequency evidenced in the present study may suggest that the specific environmental sound categories used in the test corpus were simply more easily separable with frequency information than with temporal cues. It is possible that other environmental sound sources, or other types of stimuli with more elaborate temporal structure than environmental textures, require more structured time representations.

While discriminating broad categories of environmental sound sources is a relevant auditory behavior for humans, other behaviors such as discriminating speech phonemes uttered by a single speaker (understanding speech), or variations of musical timbre by a single instrument (playing the violin) may have been more important driving-forces in the development of our auditory representations, and therefore more likely to reveal more extensive use of the rate and scale physical dimensions. For speech in particular, certain phonemes are well discriminated along the rate dimension (e.g., front/closed vowels corresponding to slower rates than other vowels, Mesgarani et al., 2007), and the present conclusion that frequency is much more important than all of the other features may not hold. However, phoneme-specific acoustic properties are typically encoded by distributed population responses in A1 which may not correspond directly to the cells' spectrotemporal tuning, but rather to the integration of multiple responses (Mesgarani et al., 2014), making it difficult to predict systematic dependencies on rate and scale. Reports of improvement of automatic speech recognition systems with STRFs are contrasted (Sivaram and Hermansky, 2008; Kollmeier et al., 2013), and may be most apparent in adverse listening conditions such as noise or concurrent speakers. (See also Patil et al., 2012, for a similar discussion of musical timbre).

Similarly, the classification task used in the present case-study does not reflect the full range of computations performed by biological systems on acoustic input. It is possible that other types of computations (e.g., similarity judgements) or, as noted earlier, other aspects of these computations (e.g., speed, compactness) could benefit from the additional representational power of rate and scale dimensions more than the task evaluated here. The trends identified here should therefore be confirmed on a larger sound dataset with more exemplars per category (Giannoulis et al., 2013) or, better yet, meta-analyzed across multiple separate datasets (Misdariis et al., 2010).

Finally, one should also note that the STRF model used in this study is linear, while auditory (and especially cortical) neurons have known non-linear characteristics. In particular, neurophysiological studies have suggested that a non-linear spike threshold can impact neural coding properties (Escabí et al., 2005). Further work should incorporate such non-linearities in the representations explored here, both to increase the biological relevance of the meta-analysis and to better understand the added computational value of these mechanisms compared to simpler linear representations.

## Author contributions

EH and JA contributed equally to designing and implementing the experiments, analysing data and drafting the present article. Author order was determined by seniority.

### Conflict of interest statement

The authors declare that the research was conducted in the absence of any commercial or financial relationships that could be construed as a potential conflict of interest.

## Footnotes

^1^The choice for the number of components *M* is a tradeoff between model flexibility (able to fit more arbitrarily complex distributions) and computational complexity (more parameters to estimate), and is heavily constrained by the amount of data available for model estimation. While optimal results for sound signals of a few minutes' duration are typically observed for *M* larger than 50, earlier work with shorter signals such as the one used here have shown maximal performance for *M*-values smaller than 5 (Aucouturier and Pachet, 2007a).

^2^The two dotted paths in Figure 3 correspond to applying GMMs or DTWs on time-series of observation in the raw (non dimension-reduced) FRS space. The dimension of this raw space being 30,976, the estimation of GMM parameters (*M* = 3 × 30,976 for means, *M* = 3 × 30,976 for diagonal covariances; total = 185,856 parameters) made little sense for time-series of only a few hundreds of observations (a bad case of the so-called curse of dimensionality). Similarly, DTW distances could not be computed at such high dimensions. While we couldn't evaluate series in the raw FRS space, both GMMs and DTW were evaluated on a dimension-reduced FRS space preserving 99.99% of its variance (upper 2 paths of the same Figure 3).
